# Liquid computing on and off the edge of chaos with a striatal microcircuit

**DOI:** 10.3389/fncom.2014.00130

**Published:** 2014-11-21

**Authors:** Carlos Toledo-Suárez, Renato Duarte, Abigail Morrison

**Affiliations:** ^1^Bernstein Center Freiburg, Albert-Ludwig University of FreiburgFreiburg, Germany; ^2^Faculty of Biology, Albert-Ludwig University of FreiburgFreiburg, Germany; ^3^Department of Computational Biology, School of Computer Science and CommunicationStockholm, Sweden; ^4^Institute for Advanced Simulation (IAS-6) and Institute of Neuroscience and Medicine (INM-6), Jülich Research Centre and JARAJülich, Germany; ^5^Institute of Adaptive and Neural Computation, School of Informatics, University of EdinburghEdinburgh, UK; ^6^Faculty of Psychology, Institute of Cognitive Neuroscience, Ruhr-University BochumBochum, Germany

**Keywords:** striatum, liquid state machine, edge of chaos, state representation, generalization

## Abstract

In reinforcement learning theories of the basal ganglia, there is a need for the expected rewards corresponding to relevant environmental states to be maintained and modified during the learning process. However, the representation of these states that allows them to be associated with reward expectations remains unclear. Previous studies have tended to rely on pre-defined partitioning of states encoded by disjunct neuronal groups or sparse topological drives. A more likely scenario is that striatal neurons are involved in the encoding of multiple different states through their spike patterns, and that an appropriate partitioning of an environment is learned on the basis of task constraints, thus minimizing the number of states involved in solving a particular task. Here we show that striatal activity is sufficient to implement a liquid state, an important prerequisite for such a computation, whereby transient patterns of striatal activity are mapped onto the relevant states. We develop a simple small scale model of the striatum which can reproduce key features of the experimentally observed activity of the major cell types of the striatum. We then use the activity of this network as input for the supervised training of four simple linear readouts to learn three different functions on a plane, where the network is stimulated with the spike coded position of the agent. We discover that the network configuration that best reproduces striatal activity statistics lies on the edge of chaos and has good performance on all three tasks, but that in general, the edge of chaos is a poor predictor of network performance.

## 1. Introduction

The striatum is the major input module of the basal ganglia; it is found in all vertebrate animals (Ericsson et al., 2011) and receives excitatory projections from the whole cerebral cortex (McGeorge and Faull, 1989; Zheng and Wilson, 2002). It is involved in multiple cognitive processes including not only motor control and planning, but also reward-modulated decision making (Kimura, 1990; Jaeger et al., 1995; Aldridge and Berridge, 1998; Hikosaka et al., 2000; Deffains et al., 2010). The circuitry underlying the latter function involves dopaminergic inervation from the substantia nigra pars compacta, with striatal cells differentially carrying information about actions to perform (Go) or not (NoGo) depending on their dopamine receptor types and projections to different parts of the globus pallidus (Balleine et al., 2007; Hori et al., 2009). This anatomical and neuromodulatory differentiation has led to attempts to model the action selection function within the conceptual framework of reinforcement learning (RL) theory (Sutton and Barto, 1998; Joel et al., 2002; Schönberg et al., 2007).

Previously it has been shown that a realistic dopaminergic error signal can drive the variant of RL known as temporal-difference (TD) learning (Potjans et al., 2011), however this modeling study relied on an artificially pre-defined partitioning of the environment into discrete RL states that were encoded as the firing rate of disjunct sets of cortical neurons. More recently, extending previous work of Doya (2000) on continuous time TD learning, Frémaux et al. (2013) successfully implemented a TD error signal over continuous time spiking representations of RL states, actions and value functions, relying on a sparse topographic (i.e., place cell like) encoding of states with narrow tuning curves and rate encoded value functions to solve navigation, acrobot and cartpole problems. However, although in some modalities the brain can rapidly develop a strong localized response to particular stimuli (Moser et al., 2008), this is unlikely to be a universal feature of sensory representation and it does not suggest an efficient way of integrating stimuli across modalities.

A more reasonable alternative for state representation is that the brain partitions the environment into states according to task features, such as landmarks or locations where a decision must be made, and in so doing reduces the representation to relevant states. This reduction is likely to yield a more efficient and realistic partitioning of the environment, compared to the traditional naïve approaches. We hypothesize that flexible environmental partitioning results from multimodal, distributed representations of environmental stimuli, impinging onto the striatum simultaneously from different cortical sources and requiring the striatal responses to develop their own internal states, which ought to reflect relevant features of the environment and be sufficiently discriminative to be used in RL. In other words: we deem it likely that an important computational role of striatal neurons in RL-based decision making, besides the relay of action related information, is the efficient processing and representation of the relevant learning states, a role that is compatible to that of the extension system in the arbitration-extension hypothesis proposed by Sarvestani et al. (2011).

How could such processing be compatible with the characteristic activity displayed by striatal neurons? Analysis of *in vivo* and *in vitro* experiments, as well as simulations of striatal activity, reveal the existence of cell assemblies which can be verified by means of clustering the medium spiny neurons according to their spike trains' correlations (Carrillo-Reid et al., 2008; Humphries et al., 2009; Ponzi and Wickens, 2010; Adler et al., 2012). However, it is not clear how such assemblies could be used to encode RL-states or indeed any RL-related variable. More generally, the computational role of this sequential episodic firing activity is not completely understood; it is present not only during the encoding and execution of motor sequences and programs, but persistent also under random or even fixed cortical excitation, i.e., does not reach a stable state. This transient dynamics led Ponzi and Wickens (2010) to claim it could be considered as an instance of metastable state switching in inhibitory networks (Rabinovich et al., 2001), known as winner-less competition (WLC).

We explore a complementary interpretation of striatal activity within the framework of another important theoretical spike-based model of real-time computation without stable states: the liquid state machine (LSM) introduced by Maass et al. (2002). An LSM relies on the capacity of the perturbed state of an excitable medium to store information of previous perturbations, analogous to the ripples generated on the surface of a pool of water when pebbles are thrown into it. Maass et al. (2002) proved that an LSM has universal computing power, in that it is possible to train linear readouts to learn a function representing a real-time analysis of the continuous input sequence of disturbances, as long as two key properties are met. The first, known as the separation property, refers to the ability to map different inputs to clearly discernible trajectories of liquid states, i.e., the distance between different network states ought to be caused by and reflect the distance between the different inputs that drove it, even when dealing with infinitesimally small differences in input patterns. The second, known as the approximation property, refers to the ability of a memoryless readout mechanism to produce a desired output based only on the network's internal states, i.e., the readouts must be capable of distinguishing the liquid states and transforming them into target outputs.

It is still poorly understood how the characteristics of a neuronal network implementation of an LSM correlate with its learning performance (Lukosevicius and Jaeger, 2009). The first neural microcircuit implementations of an LSM exhibited a connectivity structure and synaptic weight distributions based on a single cortical microcolumn (Maass et al., 2004). A model of cerebellar circuitry with LSM properties has also been proposed (Yamazaki and Tanaka, 2007). However, these findings cannot be assumed to generalize to the striatum, which is a purely inhibitory network with weak recurrent connections and low firing rates (Miller et al., 2008). These characteristics do not make a striatal microcircuit an obvious choice for the implementation of an LSM. With an average firing rate for the medium spiny neurons (MSNs) of around 5 spikes/s, it is a challenge to understand how they could support a measurable separation in activity for different inputs that can be maintained during quiescent periods where the neuron hardly fires. The challenge is enhanced in the case of a purely inhibitory network, as each additional spike can only reduce the activity in the network. So far, there has been neither formal nor practical demonstration of LSM properties for a model of the striatal microcircuit.

However, evidence exists that provides some hope to counter this somewhat unpromising outlook. Ponzi and Wickens 2012 demonstrated that under certain connectivity conditions a network of MSN neurons was able to generate different responses to different stimuli. This suggests that the network potentially possesses the separation property necessary to implement an LSM. Indeed, the authors of that study later speculated that the transient dynamics of the network could be a substrate for reservoir computing (Ponzi and Wickens, 2013). Moreover, the weak recurrence in the network facilitates dynamics that spreads on the timescale of seconds (Carrillo-Reid et al., 2008), which could enable the fading memory of previous inputs necessary for liquid computing, but it is not clear if this mechanism can compensate for the sparse and inhibitory activity.

In order to address the question of the suitability of striatal activity for liquid computing, we develop and investigate a small scale model of the striatal microcircuit consisting of both medium spiny neurons and fast spiking interneurons (FSIs) (Section 2.1). We first demonstrate that our model can reproduce key statistical features that have been experimentally observed for each neuron type, to ensure that our examination of the properties of the network activity takes place in a biologically relevant regime. We then show that these activity statistics are compatible with fulfilling the separation property (Section 2.3), whereby an infinitesimal difference in inputs results in separable network states. We assess the approximation property in terms of the ability of linear read-out neurons to extract information from the network state. To do so, and simultaneously provide a concrete demonstration of liquid computing, we train four linear read-out neurons on the low-pass filtered activity of the proposed striatal microcircuit to learn three different target functions, formalized as the motion of an agent on a flat, 2*D* surface (Section 2.4). Our results show that the transient dynamics of a purely inhibitory neuronal network at low rates can indeed perform liquid computing.

Previously, the connectivity of MSN networks was found to be a crucial factor in establishing rich transient dynamics and sensitivity to varying stimuli (Ponzi and Wickens, 2012, 2013). We therefore systematically investigate how the performance, the sensitivity to perturbations, and the activity statistics of the neuronal network depend on the strength of the connection from the cortical input to the striatal microcircuit and on the strength of the recurrent connections within the microcircuit. Our main finding is that the network configuration that most accurately reproduces striatal activity statistics is well suited to solving the tasks. Whereas alternative configurations can be identified that perform better on individual tasks, no network configuration achieved consistently higher performance across all the tasks. Finally, in Section 3 we examine the limits of our approach and the implications of an LSM interpretation of striatal activity for future experimental and modeling studies.

## 2. Results

### 2.1. Striatal microcircuit

**Figure 1:**
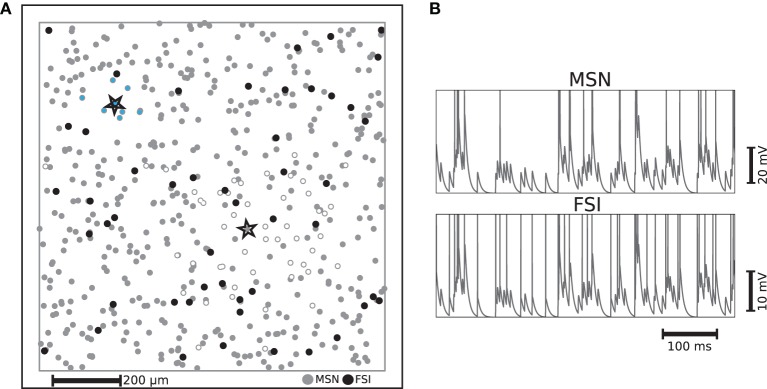
**(A)** Example of distribution of striatal neurons used as the substrate for a liquid state machine on a 1mm × 1mm plane with periodic boundaries. Each FSI is connected to neighboring MSNs on a fixed radius with a fixed probability of 74% and each MSN is connected to neighboring MSNs with a Gaussian distribution, such that the probability of making a synapse a standard deviation away is fixed to 20%. The MSN and FSI units enclosed by stars are example neurons whose synaptic targets are indicated by MSNs filled with white and light blue respectively. **(B)** Example membrane potential trajectories for the two kinds of striatal neurons implemented.

Throughout this study, we investigate the properties of a simple striatal microcircuit (illustrated in Figure 1A) and its suitability to act as a substrate for a liquid state machine. In many features of the model we aimed for biological realism and based our choices on various sources of experimental data, namely in the proportional representation of both major striatal cell types, their connection probability and the numbers and strengths of connections between populations of cell types (Kawaguchi et al., 1995; Oorschot, 1996; Koos et al., 2004; Planert et al., 2010). Some other aspects of the model, however, require simplifications to reduce the overall complexity of the model and the number of free parameters to be tuned. This is the case with the morphological and biophysical properties of the different cell types, i.e., we use point neurons and do not discriminate between striosome/matrisome cells. Nevertheless, taking all these extra details into account wouldn't provide much additional insight into the computational features investigated here.

Medium spiny neurons (MSNs), which form sparse recurrent inhibitory synapses (Tunstall et al., 2002; Planert et al., 2010), account for at least 90% of the striatal neurons (Oorschot, 1996). This suggests that they should be seen as an indispensable component of any function ascribed to this subcortical structure. The majority of the remaining neurons found in the striatum, which provide a strong feed-forward inhibition to a considerable fraction of those MSNs throughout the range of their axonal arborization, are GABAergic fast spiking interneurons (FSIs). Thus, despite being largely outnumbered, FSIs exert a non-negligible influence on MSNs (Kawaguchi et al., 1995; Koos et al., 2004).

Our microcircuit comprises 500 medium spiny neurons (MSNs) and 50 fast spiking interneurons (FSIs) with locations drawn from a two dimensional uniform distribution on a 1mm × 1mm plane with periodic boundaries, resulting in a network approximately a factor of 10 less dense than biological striatal networks. However, anatomical findings suggest that striatal neurons in close proximity share few cortical inputs (Zheng and Wilson, 2002). This allows us to abstract the influence of the remaining neurons, without loss of biological plausibility, as stochastic input to the modeled neurons, given that those remaining neurons would probably not be functionally involved with the same task. The ratio of 10 MSNs to every FSI corresponds to the experimentally observed proportion (Kawaguchi et al., 1995). The two neuron types are implemented using the multi-timescale adaptive threshold neuron model (Kobayashi et al., 2009) tuned for intrinsic bursting for MSNs (Aldridge and Gilman, 1991) and fast spiking for FSIs, with parameters taken from the literature (Tunstall et al., 2002; Koos et al., 2004; Gertler et al., 2008). The different characteristic activity patterns are illustrated in Figure 1B, which shows representative examples of membrane potential and firing activity for both neuron types, each receiving a direct current whose value is 1 pA less than their respective rheobase currents and an identical excitatory Poissonian train at a rate of 150 spikes/s. The activity statistics of the two neuron types in the microcircuit model are examined in greater detail in Section 2.2.

The connectivity structure we implement in this model is based on and consistent with striatal slice experiments that (similarly to our case) did not discriminate between striosome/matrisome cells. These studies revealed that the inhibitory projections of the MSNs are sparser and spread over a larger area than those of the FSIs (Koos et al., 2004; Planert et al., 2010). We incorporate these findings (particularly those of Planert et al., 2010) in the specification of the connectivity structure of the striatal microcircuit: each FSI is connected to neighboring MSNs within a fixed radius of 100 μm with a fixed probability of 74%, whereas each MSN is connected to neighboring MSNs according to a Gaussian distribution, such that the probability of making a synapse to a neuron one standard deviation away is fixed to 20%. The strengths of each type of synaptic connection are selected from a uniform distribution within realistic ranges (Koos et al., 2004). The FSIs receive no recurrent input from MSNs (Bennett and Bolam, 1994), and gap junctions between FSIs have not been incorporated since there is no strong evidence for them to synchronize FSIs' firing (Berke, 2008). Each MSN and FSI receive input from a fixed 25% of randomly selected cortical neurons. For a description of the input encoding features of these cortical neurons, see Section 4.1; a complete listing of our model dynamics, connectivity and parameters can be found in the Supplementary Materials.

### 2.2. Activity statistics of striatal microcircuit model

In all experiments carried out in this study, the synaptic weights are multiplied by scale factors, determined by the type of connection: the factor *w_s_* scales all intra-striatal synapses, and the factor *w_c_* scales all synapses between the cortical input neurons and the striatal neurons. Synaptic weights between cortical neurons and FSIs are subject to an additional constant scale factor, to account for the observed higher sensitivity of these neurons to cortical input compared to MSNs (Parthasarathy and Graybiel, 1997).

**Figure 2:**
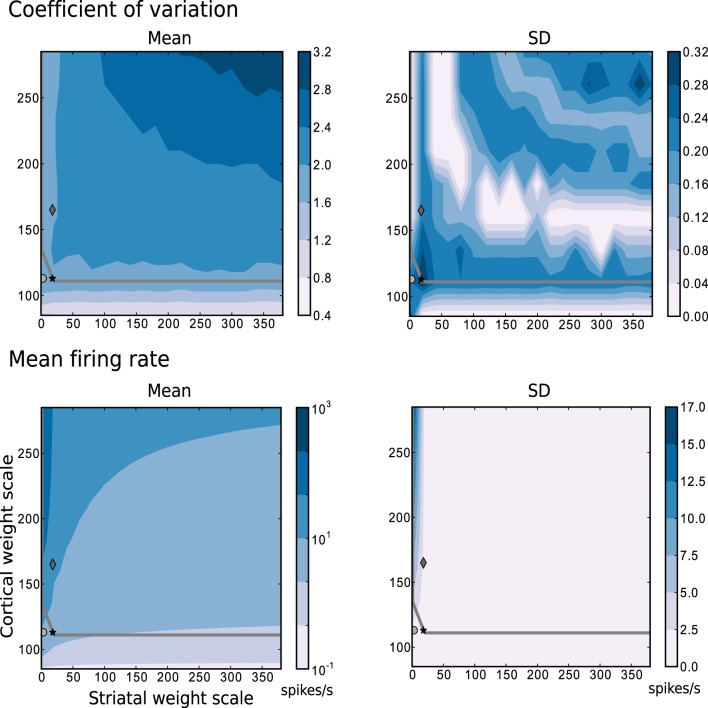
**Mean and standard deviation of the mode of the coefficient of variation and the mean firing rate as functions of the scale factors for the intra-striatal synapses *w*_s_ and the corticostriatal synapses *w*_c_**. Statistics are calculated from 10 realizations of each network configuration whilst following random trajectories on the flat surface. The region of chaotic behavior is located above and to the right of the gray overlayed curve. Markers indicate configurations of the network identified for further analysis: black star: {*w*_s_ = 18, *w*_c_ = 113}, dark gray diamond: {18, 165}, light gray circle: {0, 113}.

The most relevant statistical descriptors of population activity (mean firing rates and coefficient of variation of the interspike intervals) are displayed in Figure 2 as a function of the synaptic scaling factors, measured according to the description in Section 4.2.2 while following random trajectories on the flat surface. The overlayed gray curve indicates the edge of chaos transition region, calculated as described in Section 4.2.3. The chaotic region in this case is located above the curve. These results reveal that a large portion of the configurations explored have a mean firing rate below 10 spikes/s, a mean CV above 2, and that the edge of chaos is a reliable predictor of the area with 1.6 < CV ≤ 2. Based on these results, we determined three interesting network configurations that we subsequently investigate in greater detail.

**Figure 3:**
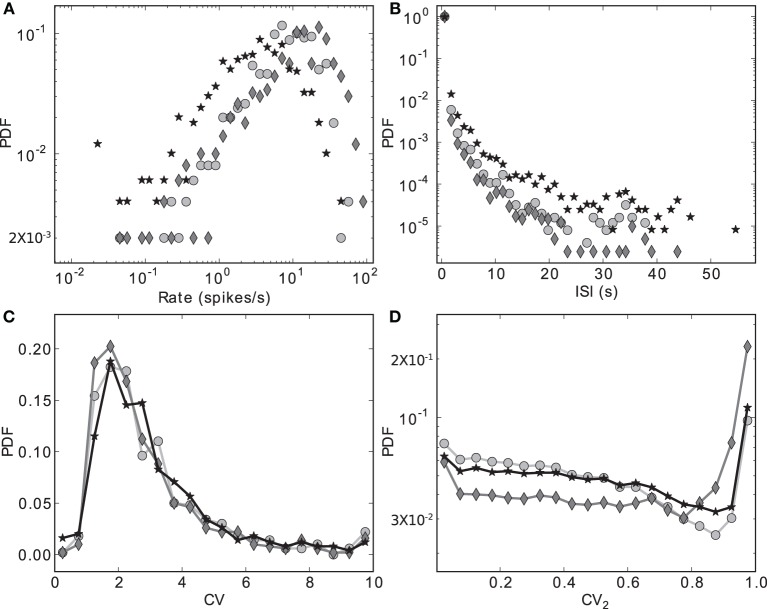
**Discrete probability density functions for activity statistics of the MSN population**. **(A)** Rate (bin size = 0.1 in logarithmic space), **(B)** Interspike intervals (bin size = 1.2 s), **(C)** Coefficient of variation (bin size = 0.5), **(D)** Local coefficient of variation (bin size = 0.05). Markers indicate different configurations of the scaling factors for the strengths of the recurrent connections within the striatal microcircuit *W*_s_ and for the connections from the cortical input to the striatum *W*_c_. Markers as in Figure 2: black star: {*w*_s_ = 18, *w*_c_ = 113}, dark gray diamond: {18, 165}, light gray circle: {0, 113}.

Figure 3 shows the distributions of four key activity statistics for the MSN population in our proposed model of the striatal microcircuit: firing rate, interspike interval (ISI), coefficient of variation (CV) and local coefficient of variation (CV_2_). Whereas the coefficient of variation is a measure of regularity in a neuron's spike train, calculated as the standard deviation of the ISIs divided by the mean, the local coefficient of variation is a measure of episodic firing. For a neuron with *N* spikes in the recording period, a series of length *N* − 1 of values for the local coefficient of variation can be calculated from subsequent ISIs, where the *n*th value is calculated as *CV^n^*_2_ = |*ISI*_*n* + 1_ − *ISI*_*n*_|/(*ISI*_*n* + 1_ + *ISI*_*n*_). The distribution of these values over all the recorded neurons is shown in Figure 3D.

The data series in Figure 3 correspond to the three different {*w_s_*, *w_c_*} pairs identified with the corresponding markers in Figures 2, **7–9**, as samples of the full parameter space, where the combination of measured variables and connectivity is more informative, to be compared among the different problems examined in Section 2.4. The condition indicated by black stars, corresponding to {*w_s_* = 18, *w_c_* = 113} accurately reproduces key statistical features of MSN activity that have been experimentally observed *in vivo* (Wilson, 1993; Miller et al., 2008). The modeled MSN population displays a broad distribution of firing rates, with a low mean of around 4 spikes/s. Additionally, the individual spike trains are highly irregular, presenting a broad and unimodal distribution of the coefficient of variation with a peak at around 2. Finally, the spike trains exhibit episodic firing patterns, which is illustrated by the distribution of the local coefficient of variation, showing a bimodal pattern with peaks near zero and one, despite the unimodality observed in the ISI distribution. In the remaining of this section, we examine other activity features of the striatial microcircuit focusing on this configuration of weight scaling factors, as it provides the best fit to the four statistics shown in Figure 3.

The other two configurations, indicated by diamonds and circles, show qualitatively similar statistics, with the important difference being that they display mean firing rates of ~ 14 and ~ 8 spikes/s respectively, which are too high to be considered as realistic MSN behavior. In both cases, firing rates below 1 spikes/s are under-represented and firing rates above 20 spikes/s are over-represented.

**Figure 4:**
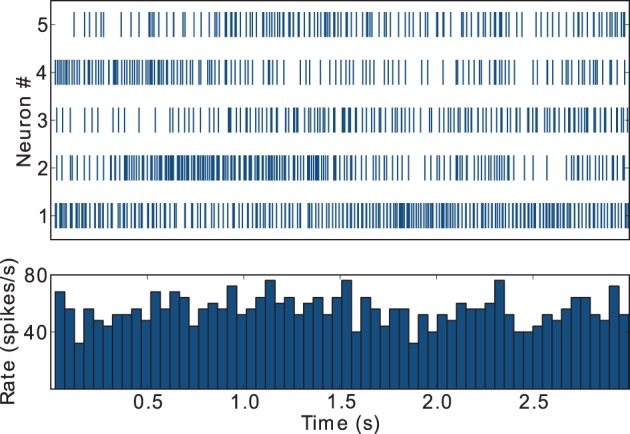
**Raster plot and activity histogram (bin = 50 ms) for the five FSIs with highest firing rates in the network with weights configuration {*w*_s_ = 18, *w*_c_ = 113}, indicated by the black star markers in Figure 2**.

During awake behavioral states, FSIs tend to display firing rates in the gamma frequency band, i.e., above 30 spikes/s, but show little or no coordinated population response to task related events such as instruction cues (Berke, 2008). This result is captured by our model FSIs, as depicted in Figure 4, which exemplifies the spike trains and activity histogram for the five FSIs that display the highest firing rates in the striatal microcircuit configured with {*w*_s_ = 18, *w*_c_ = 113} (black star markers in Figure 3). These FSIs fire consistently in the gamma band and lack coordinated behavior during task performance, in agreement with the supra-cited experimental findings.

As an additional assessment of the firing rate behavior for the striatal microcircuit with the {*w*_s_ = 18, *w*_c_ = 113} configuration, we examine the percentages of inter-spike intervals for both the MSN and FSI populations that fall into each of the classical ECoG frequency bands. Table 1 shows that the ISI percentages generated by our model are a good fit to the relative contributions of frequency bands to the firing rate distributions observed experimentally by Schulz et al. (2011).

**Table 1:** **Percentage of ISIs according to classical ECoG frequency bands for neurons in the striatal microcircuit configured with {*w*_c_ = 18, *w*_s_ = 113} (black star markers in Figures 2 and 3)**.

**Frequency band ISIs**	**Slow and delta, <4 Hz > 250 ms**	**Theta-alpha, 4-12.5 Hz 250–80 ms**	**Beta, 12.5-33 Hz 80–30 ms**	**Gamma, >33 Hz <30 ms**
MSN (*n* = 500)	76.8 ± 12.4 %	14.6 ± 6.8 %	6.5 ± 4.6 %	2.1 ± 2.2%
[*n* = 68]	[53.3 ± 28.2 %]	[16.2 ± 14.2 %]	[19.3 ± 16.4 %]	[11.1 ± 15.0 %]
FSI (*n* = 5)	34.5 ± 3.6 %	15.1 ± 4.9 %	16.1 ± 2.2 %	34.2 ± 9.2%
[*n* = 9]	[29.9 ± 30.0 %]	[4.9 ± 3.4 %]	[21.6 ± 12.6 %]	[43.6 ± 32.8 %]

*Values in brackets taken from Schulz et al. (2011) for comparison*.

In summary, the results described in this section demonstrate that despite being a small network of point neuron models, our proposed model of the striatal microcircuit can account for and accurately reproduce many experimentally observed statistical features of MSN and FSI spiking behavior, both on the level of the individual spike trains and on the level of population activity. We can thus safely assume that the dynamics displayed by this model are sufficiently representative of striatal activity to allow us to use this model circuit to investigate tentative computational properties which may be realized by striatal firing patterns.

### 2.3. Separation property

To be a substrate for a liquid state machine, a network must satisfy the separation property, as demonstrated by Maass et al. (2002). In other words, trajectories of internal states evoked by two different input streams must be sufficiently discernible to maintain or amplify their separation. It has been previously shown that different input stimuli to a spiking neuronal network model of MSNs can evoke firing in different cell populations (Ponzi and Wickens, 2012). However, in order to demonstrate the separation property, we must show that stimuli that are infinitesimally different in input space (for example, with one single spike shifted) generate discernibly different network responses.

To this end, we stimulate the cortical input layer to the striatal microcircuit according to a randomly chosen agent position *x* (see Section 4.1 for details), and then repeat the experiment from the same initial conditions with the agent positioned at *x* + ϵ, i.e., a small distance ϵ away from the originally chosen position. The Euclidean distance between the low-pass filtered activity of the cortical neurons representing the first and the second position serves as a measurement of the input separation (see Section 4.2.1).

**Figure 5:**
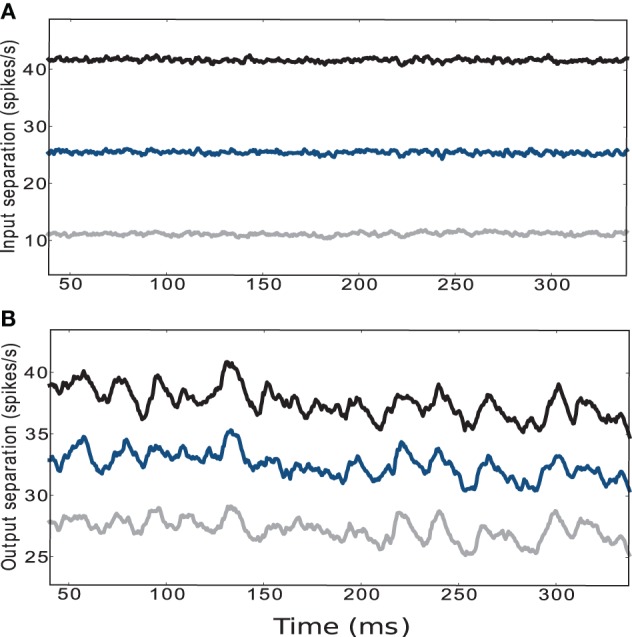
**(A)** Euclidean separation of the low-pass filtered cortical input activity. **(B)** Output activity of the striatal microcircuit with weight scaling factors configured as {*w*_s_ = 18, *w*_c_ = 113} (black star markers in Figure 2). Curves are averaged over 25 samples consisting of 5 different initial conditions and circuit instantiations, where each network configuration receives 5 different pairs of sets of input trains corresponding to the encoding of 5 randomly chosen pairs of positions. The distance between the pairs of positions is set to a hundredth (gray), a tenth (blue) and a fifth (black) of the environment's width.

The traces displayed in the upper panel of Figure 5 refer to the average Euclidean distance between the input trajectories for five different initial conditions and circuit instantiations and five different pairs of positions with fixed ϵ. The configuration of weight scaling factors to and within the striatal microcircuit are set to {*w*_s_ = 18, *w*_c_ = 113}, i.e., providing the most faithful fit to experimentally observed activity statistics as described in the previous section. The lower panel of Figure 5 shows the average Euclidean distance of the low-pass filtered striatal microcircuit output activity evoked by the corresponding input activities described above, calculated according to Equations 1 and 2, as explained in Section 4.2.1. Although the curves are noisier, the activity of the striatal microcircuit clearly maintains the separation of its inputs, even when the separation in the input activity is driven by a difference in agent position of one hundredth of the environment width. We conclude that despite the low firing rates and the purely inhibitory character, the activity of the striatum is indeed sufficiently rich to support adequate state separation, even in response to infinitesimally small differences at the input level.

### 2.4. Computational properties of the striatal microcircuit model

To assess the computational capabilities of the proposed striatal microcircuit as a liquid state machine, we chose three target functions represented as trajectories on a 2*D* plane. Note that the functions chosen are not intended to explicitly model navigation; the interpretation of the functions to be learned as trajectories on a plane is simply to ease visualization. To evaluate the performance, the striatal microcircuit is connected to four linear read-out neurons that encode the four cardinal directions on the plane. During the training phase, the strengths of the synapses between the striatal neurons and the read-out units are learned according to a supervised learning algorithm; see Section 4.3 for a detailed description of the learning process and interpretation of the activity of the read-out neurons. The use of a supervised learning algorithm in this context is not intended to reflect our assumptions about the downstream processing of striatal activity in the rest of the basal ganglia and does not influence the dynamics of the striatal network. It simply serves to demonstrate that network states can be separated and to determine to what extent the transient responses of the striatal microcircuit are sufficiently informative to be used as the basis for learning.

In this framework, we investigate the second feature necessary for a system to implement a liquid state machine, the approximation property. We start by determining the ability of a set of linear readout units to use the network responses to produce a desired target output, i.e., to associate them with a target action (exemplified by the motion of an agent along a surface, following a specific path). The approximation property is thus assessed as the performance of these readout units (see Section 4.4.1).

Another property that is important for the hypothesized role of the striatum in providing a partitioning of the environment into relevant states, is that similar input stimuli can be be mapped to the same action. To evaluate this, we introduce a measure of generalization (see Section 4.4.2). Having been trained on a variety of points in input space, we assess how well the system can map nearby points onto the corresponding actions.

**Figure 6:**
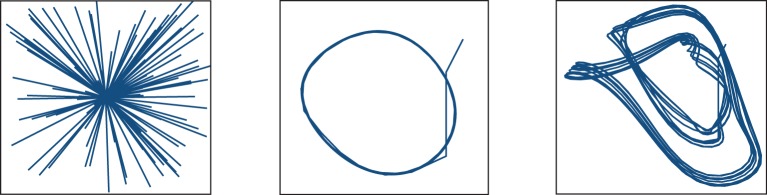
**Functions to learn: straight trajectories toward a goal at the center of the surface (left), and following a discretized version of the delay Mackey-Glass differential equation with delays of τ = 5 (center) and τ = 15 (right)**.

Figure 6 shows the three target functions we used to illustrate these principles: straight trajectories toward a goal at the center of the surface (left), and discretized versions of the Mackey-Glass differential equation (Farmer, 1982):

$$\begin{array}{l} \Delta y_{i}=\alpha x_{i}{\left(1+{\left(x_{i}/\eta \right)}^{\gamma }\right)}^{-1}-\beta y_{i}\\ \Delta x_{i}=y_{i-\tau }-x_{i} \end{array}$$

with α = 0.2, β = 0.1, γ = 10 and η = 15 and τ can assume two different values, setting a different task complexity: τ = 5 (center) and τ = 23 (right).

The function of approaching a goal in straight trajectories was chosen as a simple way of implementing and exploring the problem of stabilizing an input to a desired state regardless of the initial conditions. The Mackey-Glass equation, on the other hand, allows us to create tasks of increasing complexity by varying the delay τ that determines the dependence of future increments on previous function values. Larger values of τ force the microcircuit to exploit its fading memory of previous inputs and constitute a more complex task.

It's important to highlight another important difference between these studied instances. Whereas for the first case we randomly reset the position from which the agent begins after a predefined number of training/testing steps, for the Mackey-Glass functions the starting position is fixed and the subsequent trajectory is generated according to the Mackey-Glass equations, for the entirety of the training and testing steps.

In the following, we investigate how the performance and generalization capacity of the linear read-out neurons of the striatal network depends on the strength of connections between the cortical and the striatal neurons, and the strength of the recurrent connections within the striatum. As individual synaptic weights are drawn from specific distributions (see Sections 2.1, 4.4.2), the strengths of the connections are expressed as scaling factors that multiply the entire distributions (*w*_c_ and *w*_s_, respectively). In Legenstein and Maass (2007), a similar analysis of the impact of connectivity parameters on performance and generalization was carried out for a 3*D* cortical microcircuit model with recurrent excitation and inhibition.

For each function, we measure the performance as the average difference between the direction indicated by the read-out neurons and the true direction according to the function to be learned. Analogously, we measure generalization capacity as the average difference between the directions indicated by the read-out neurons for positions learned during the training and the directions indicated for near-by positions. The calculation is given in greater detail in Section 4.2.2.

#### 2.4.1. Straight trajectories toward a goal

**Figure 7:**
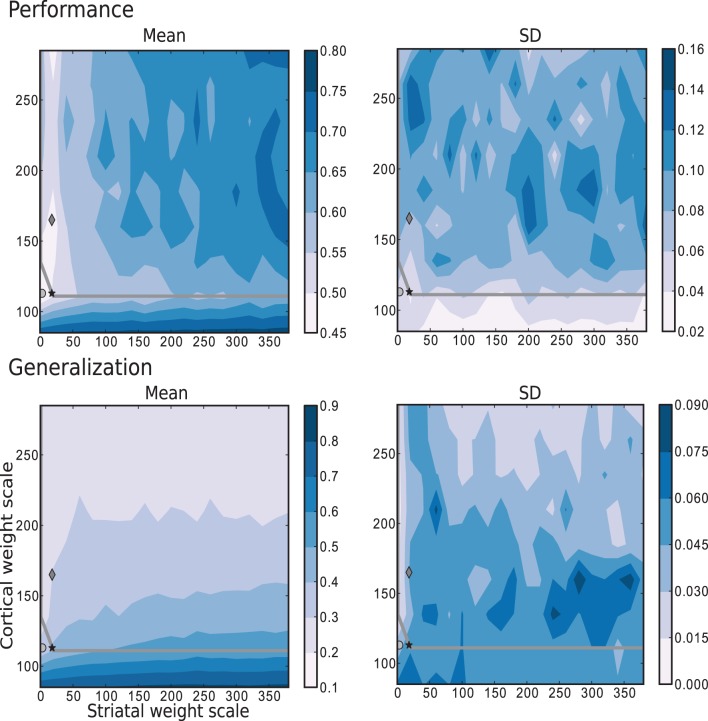
**Mean and standard deviation of the performance and generalization capabilities achieved by the linear readouts for learning straight trajectories toward a goal**. The region of chaotic behavior is located above and to the right of the gray overlayed curve. Statistics are calculated from 10 realizations of each network configuration. Markers correspond to those used in Figure 2.

The results depicted in Figure 7 show the performance and generalization capacities achieved by using four linear readouts connected to the microcircuit for the simplest task, learning straight trajectories toward a goal. Lighter colors denote better performance and generalization capacity, and the overlayed gray curve indicates the edge of chaos transition region, calculated as described in Section 4.2.2. The chaotic region in this case is located above the curve.

The best performance on this task is achieved by networks with weak or even absent intra-striatal synaptic connections (*w*_s_ ∈ [0, 30]) and moderate corticostriatal synaptic strengths. Networks with stronger corticostriatal connections have a greater capacity to generalize from learned positions to near-by positions; to a lesser extent networks with very weak intra-striatal connections display a better generalization capacity in the present task than those with stronger intra-striatal connections.

These results raise the question of whether the existence of the inhibitory intra-striatal connections is at all beneficial for solving this task. For over half of the investigated values for the corticostriatal weight scaling factor *w*_c_, the best performing circuit is found for *w*_s_ > 0. However, for the best performing networks overall, including the configuration with the most realistic MSN and FSI firing statistics (black star marker), there is no improvement in performance for a recurrently connected network with respect to an unconnected one (with *w*_s_ = 0, corresponding to the results depicted in the first column of each figure).

A small portion of the area where generalization is highest coincides with the area of highest performance, but overall good generalization is not a good indicator of high performance, and vice versa. Similarly, while the best performing networks lie on or near the edge of chaos, it is not a consistently good predictor of either performance or generalization.

#### 2.4.2. Mackey-glass functions

As previously mentioned, using the Mackey-Glass function to generate the target trajectories allows us to control the task complexity. Particularly, by manipulating the delay parameter τ, we can control the amount of memory necessary to solve the task, which amounts to the fading memory property of liquid state machines.

**Figure 8:**
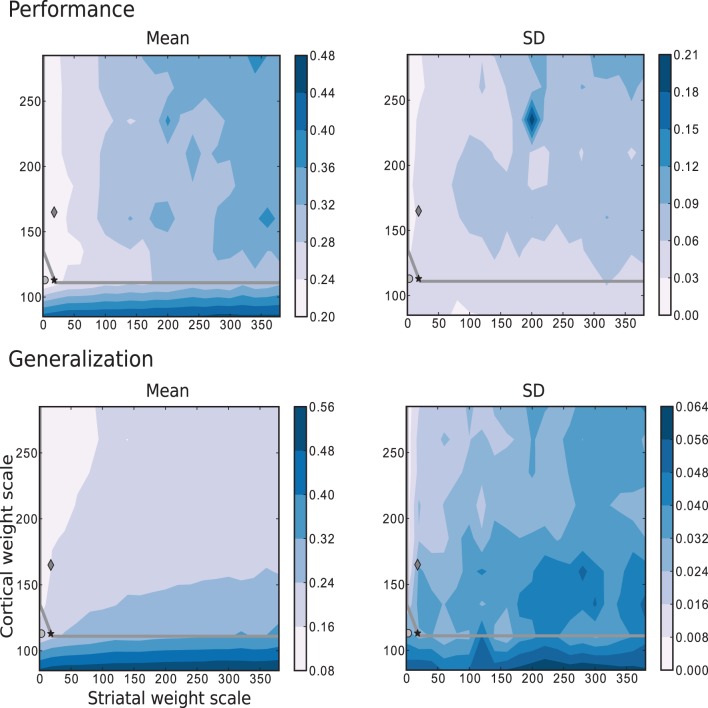
**Mean and standard deviation of the performance and generalization capabilities achieved by the linear readouts for learning the discretized Mackey-Glass function with τ = 5**. Statistics are calculated from 10 realizations of each network configuration. Markers and overlayed gray curve as in Figure 2.

The simplest scenario, with τ = 5 is depicted in Figure 8, which shows the performance and generalization capabilities of the four linear readouts connected to the microcircuit as a function of the connection scale parameters. Unlike the previous task, the best performing circuits are found in the absence of recurrent striatal connections (*w*_s_ = 0). In the presence of recurrent inhibition (*w*_s_ > 0), the circuits perform, at best, as well as unconnected networks. Additionally, the highest generalization capacity coincides with the highest performance for low values of *w*_s_. In contrast with the previous task, the great majority of the network configurations with the highest performance are to be found in the chaotic regime, with a smaller area on or below the edge of chaos.

**Figure 9:**
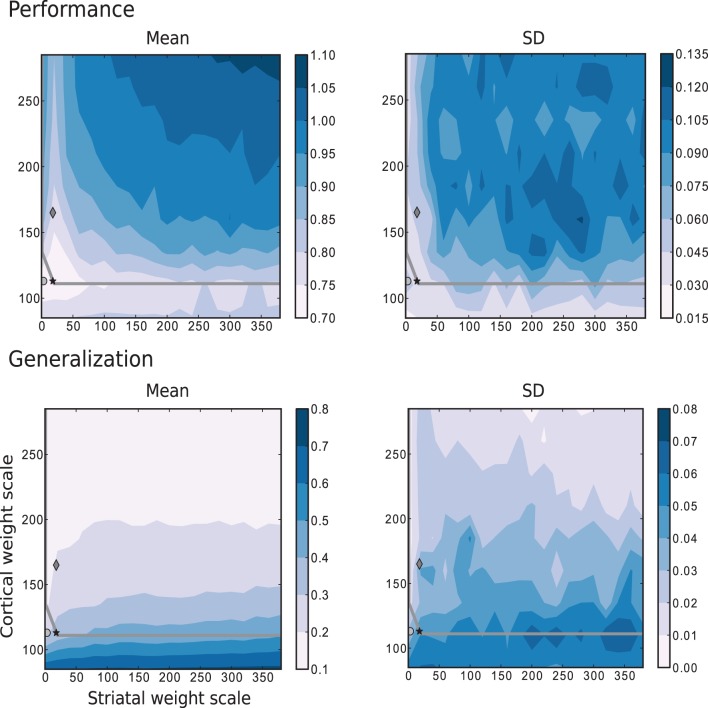
**Mean and standard deviation of the performance and generalization capabilities achieved by the linear readouts for learning the discretized Mackey-Glass function with τ = 15**. Statistics are calculated from 10 realizations of each network configuration. Markers and overlayed gray curve as in Figure 2.

Figure 9 corresponds to the results obtained for the same parameter space using the more complex task where the trajectories are generated by the Mackey-Glass equation with τ = 15. These results show that for more than two thirds of the investigated values for the corticostriatal weight scaling factor *w*_c_, there are recurrently connected circuits that have higher performance than unconnected ones. However, the network configuration that produces the most realistic MSN and FSI firing statistics performs no better than network with the same corticostriatal weight scaling factor *w*_c_ but no recurrent connections. Unlike the previous tasks, there is no overlap between the area with highest performance (low *w*_c_ and low *w*_s_) and the area with the best generalization (high *w*_c_). Additionally, the region corresponding to the edge of chaos, does not correlate with either performance or generalization, as there are approximately as many network configurations giving the highest performance above the edge of chaos as below it, leading to the conclusion that the edge of chaos itself is a poor predictor of performance.

#### 2.4.3. Comparison of performance on different tasks

**Figure 10:**
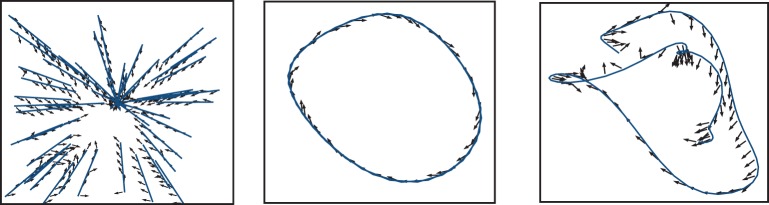
**Normalized increments, indicated by black arrows, advocated by the microcircuit after training for the three functions, using the network configuration {*w*_s_ = 18, *w*_c_ = 113} (black star markers in Figure 2)**.

Comparing the performance of the network in Figures 7–9 indicates that the easiest function for our model is the Mackey-Glass with τ = 5 (MG5), followed by the one of approaching in straight trajectories toward a goal (GOAL), and the most difficult is the Mackey-Glass function with τ = 15 (MG15). This can be seen also in Figure 10, which shows examples of the normalized increments, indicated by black arrows, advocated by our microcircuit after training for the three functions, using the best combination of connectivity parameters that most accurately replicates striatal activity statistics, as indicated by the black star marker in Figure 2 and discussed in Section 2.2. The incremental performance achieved in the different tasks is easily verified by the alignment of the advocated actions in Figure 10 with the corresponding target trajectories. Arrows are more aligned with the function for problem MG5, followed by GOAL, and less aligned for MG15, particularly in regions where the trajectory crosses itself. In these areas, the correct action can only be discriminated using the information from previous values, forcing the microcircuit to exploit its fading memory of previous inputs.

For the easiest task explored, there seems to be no benefit of having intrastriatal recurrent connectivity to the network's representational capacity; networks with *w*_s_ = 0 perform as well as or better than networks with *w*_s_ > 0 for any given choice of *w*_c_. However, as the tasks become more complicated, the area of best performance shifts to network configurations with *w*_s_ > 0, as can be seen in Figures 7, 9. It is also worth noting that higher performances are not linked to high firing rates (by comparing with Figure 2). Neither of the two other network congurations (Dark gray diamond {*w*_s_ = 18, *w*_c_ = 165} and light gray circle {0, 113}) do a consistently better job than the network configuration indicated by the black star marker {18, 113} although they have similar activity dynamics at higher rates (~ 14 and ~ 8 spikes/s respectively, compared to ~ 4 spikes/s). In general, accurate representations and the consequent best readout performance is found only over a small proportion of the explored parameter space, marked by low intra-striatal synaptic weight scales. Furthermore, it becomes impossible to find non-chaotic dynamics as the synaptic weights between cortical and striatal neurons increase, and generalization becomes less predictive of performance as the problem difficulty increases.

## 3. Discussion

This study provides the first demonstration that a purely inhibitory network with weak recurrence and low firing rates nevertheless generates activity patterns of sufficient richness to fulfil the separation and approximation properties that are the necessary and sufficient conditions for liquid computing. Throughout this study we have also practically demonstrated that the transient activity patterns of a simple striatal microcircuit can adequately represent the motion of an agent along specific paths in a 2*D* plane. These representations were shown to be informative enough to train a set of linear readouts to adequately realize a variety of generic target functions, advocating the correct actions in order to reproduce the desired trajectories.

The proposed microcircuit is shown to capture key properties of real striatal networks by using different configurations of the same neuron model to represent the MSN and FSI populations and biologically motivated connection probabilities between the populations. The model, while remaining simple, is thereby capable of reproducing several experimentally observed statistical features of MSN activity (distributions of rates, ISIs, coefficients of variation and local coefficients of variation) and FSI activity (firing rates and low synchrony), to a degree comparable to a previous study (Ponzi and Wickens, 2010) with the important addition that in our case these can be directly linked to the performance on a set of concrete learning tasks based on striatal activity.

Our results also demonstrated somewhat surprising and counter-intuitive relations between the measured properties. The capacity to generalize beyond the training data seems to be negatively correlated with the mean firing rate and the region of transition from ordered to chaotic activity: as the driving input becomes stronger and the firing rate increases, small perturbations acquire more influence and generalizing becomes more difficult. It remains unclear whether generalization could be used as a predictor of performance in general, which is especially unclear for regions of the explored parameter space that displayed higher performances. What appears to be a consistent result in our study is that generalization capacity is negatively correlated with low performance for large values of intrastriatal synaptic strengths.

Additionally, although the best network configuration with respect to activity statistics (the one that most accurately reproduces the biological system) is found on the edge of chaos, proximity to this transition regime is not a reliable predictor for high performance or generalization. The only metric that seems to significantly correlate with it is the behavior of the coefficients of variation of the interspike intervals.

These observations show a clear difference in the expected relationship between performance, generalization and the edge of chaos for a liquid state approach to striatal functioning, with respect to a similar one applied to generic cortical microcircuits (Legenstein and Maass, 2007). In the latter case it is possible to identify at first sight a clear coincidence between the edge of chaos and the zones of highest performance throughout the whole parameter space explored, and generalization could be linked to performance by subtracting a network measure from it, i.e., the kernel-quality. In our case, the transitions are more variable and it is not clear how the subtraction of a single network measure could explain the changes in performance observed when learning different functions, strongly suggesting that the capacity to generalize beyond the training data is highly dependent on the characteristics of the problem being solved.

The last observation also applies to the relationship between the edge of chaos and performance. To date there is no general proof of the significance of the edge of chaos regime for computational performance on any problem, except for the case of a generic computational task involving the discrimination of precisely timed spike patterns, assumed by the authors to be representative of the general computational capabilities of a cortical microcircuit (Legenstein and Maass, 2007; Schrauwen et al., 2009). Our results, on the other hand, may favor an alternative interpretation, such as that defended by Mitchell et al. (1994), who pointed out the importance of not claiming a generic relationship between performance of computational systems on specific problems and measures of chaotic behavior.

Our results also support the hypothesis that basal ganglia activity does not differ depending on the generalization requirements of the learning task at hand (Seger, 2008), as there is neither a consistent nor positive correlation between generalization and performance on most of the circuit configurations explored for the learning problems used. This tendency is confirmed experimentally by studies that compare tasks differing only in their generalization requirements.

A central assumption of our study was that the striatum is involved in processing cortical input in a manner that allows the relevant states to be represented within striatal microcircuits as transient, spatiotemporal activity patterns, and associated with corresponding actions. If this assumption is true, a similar degree of discrimination between different states should be achieved in the real system as it was in our reduced, simplified model of the striatum. This would allow, for example, the use of simple supervised learning algorithms to be applied to *in vivo* striatal multi-unit recordings to predict the action taken by an organism in a forced choice task (Mehring et al., 2003), which reduces to a classification of state representations developed within the striatal microcircuit.

By demonstrating the suitability of striatal microcircuits to function as a liquid state machine, particularly their ability to transform cortical input into discernible striatal activity states, we are providing the first steps toward a more realistic and comprehensive understanding of the role of basal ganglia in RL. It is reasonable to hypothesize that the manner in which these states are used by the downstream circuitry relies on dopamine-mediated learning (Potjans et al., 2011), however, we do not inquire here about the neural correlates or system level functional role of such tentative mechanisms within the basal ganglia (Sarvestani et al., 2011). Even though the scope of this study is narrow and limited to the nature of useful state representations within striatal microcircuits, it is important to realize that the ability to partition the environment into useful RL states, without resorting to artificial means and relying solely on known biophysical properties of the striatum is necessary to develop more accurate and plausible models of the nature of RL in the basal ganglia.

Computation on chaotic regimes is compatible with the presumed involvement of the striatum in the generation of random exploratory switching between motor sequences, regardless of the actual cortical input (Barnes et al., 2005). Ponzi and Wickens (2010) claimed that the cell assembly behavior found in their MSNs network simulations is an instance of chaotic switching between metastable states. In the future we intend to analyse the relationship between transient dynamics as instantiated by our liquid state approach with such behavior, and to investigate how the supervised learning used here could be replaced by a learning framework that can be linked to experimentally observed processes taking place in the basal ganglia, such as those based on error signals that reflect the role of dopamine in the striatum.

## 4. Materials and methods

This section presents a description of the peripheral constituents of the striatal liquid state machine model, the learning algorithm used to train the linear read-out neurons, and the analysis methods used to investigate network performance and dynamics.

### 4.1. Input encoding

**Figure 11:**
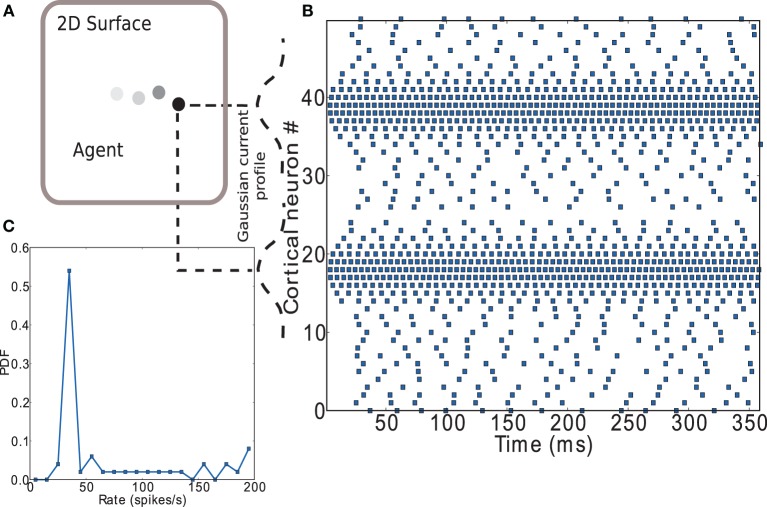
**Encoding of input to striatal microcircuit**. **(A)** Depiction of agent moving along a trajectory on a plane. **(B)** Raster plot of the cortical neurons' activity encoding position on the (arbitrary) X axis (neurons 1 to 25) and Y axis (neurons 26 to 50) of the plane. Gaussian current profiles indicate the relative magnitude of input current to cortical neurons. **(C)** Firing rate distribution for the spike trains shown in **(B)**.

In this article we investigate the ability of an inhibitory network with sparse firing to learn functions on a plane (see Figure 11A). As we are concerned with the performance of the striatal microcircuit to learn generic tasks rather than explicitly spatial tasks, the option of using a model based on grid or place cells (Moser et al., 2008) was discarded in favor of a simple way to represent two-dimensional input.

Figure 11B shows a raster plot that demonstrates the input encoding chosen: the position on each of the two axes is coded by the activity of a group of leaky integrate-and-fire neurons with exponential post-synaptic currents distributed along each axis. Each neuron receives an independent excitatory Poissonian background input at the same rate and a weak direct current with Gaussian magnitude having its maximum on the position. A complete listing of parameters used can be found in the Supplementary Materials. Figure 11C shows the firing rate distribution for the spike trains shown in Figure 11B. A large range of firing rates has been found in lateral intraparietal area (LIP) of cortex (O'Leary and Lisberger, 2012). LIP is an input area to striatum (Saint-Cyr et al., 1990) which has been hypothesized to act as an input saliency map with strength of salience represented by activity level (Bisley and Goldberg, 2010). The range of firing rates observed in our model is somewhat higher, but the number of input neurons used is small. An equivalent effect of the input on the network could be achieved by increasing the number of input neurons while decreasing the strength of the direct current, thus decreasing the maximum rate of the input neurons; for the sake of simplicity we opt for the smaller system.

### 4.2. Activity analysis methods

In this section, we describe the methods used to analyse the circuit's spiking activity.

#### 4.2.1. Low pass filtering and euclidean separation

The low pass filtering of spike trains used for the training of linear readouts is calculated as the following sum of exponentials:

(1) $$\frac{ds_{j}}{dt}=-\frac{s_{j}}{\tau_{0}}+\frac{1}{\tau_{0}}\Sigma_{t_{j}^{f}}\delta \left(t-t_{j}^{f}\right)$$

where *j* is an index running over the striatal neurons, *t^f^_j_* are the firing times of neuron *j* and τ_0_ the exponential decay. The Euclidean distance or separation between the network states evoked by inputs *u* and *v* at time *t* is calculated as:

(2) $$\varsigma_{u,v}\left(t\right)=\sqrt{\sum_{j}{\left(s_{j}^{u}(t)-s_{j}^{v}(t)\right)}^{2}}$$

To determine the separation at the input level, the calculation proceeds analogously, replacing striatal spike trains by cortical ones.

#### 4.2.2. Activity statistics

In order to quantify the characteristics of network activity observed in the striatal microcircuit in a manner that allows a proper comparison with the relevant experimental data, we resort to the following metrics:

***Mean firing rate***. We obtain the firing rate of each MSN by dividing its total number of spikes by the simulation time, and then calculate the mean and standard deviation over all such cells. These values are then averaged over 10 network realizations per condition.

***Coefficient of variation***. We obtain the CV for the spiking activity of each MSN by dividing the standard deviation of its interspike intervals by its mean. The distribution is obtained by binning the values with a bin size of 0.5, as depicted in Figure 3. We then calculate the mode of the distribution on each circuit realization and the mean and standard deviation of the mode over 10 network realizations.

#### 4.2.3. Edge of chaos

To determine the region of transition from stable to unstable circuit dynamics, i.e., the “edge of chaos,” we perform a simple perturbation analysis, measuring the sensitivity of the network dynamics to a small perturbation. To do so, we calculate the Euclidean distance between the low-pass filtered activity of the striatal microcircuit when the agent remains in one position and that obtained at the same position and initial conditions but with one additional input spike. After smoothing this measure by applying a moving average, we define the edge of chaos as the frontier in parameter space where its natural logarithm is greater than zero after 200 ms and stays so for the remainder of the simulation.

### 4.3. Linear readouts

We implement readouts of the low-pass filtered output of the striatal microcircuit using four perceptrons (Rosenblatt, 1958), each encoding movement in one of the four cardinal directions on the 2D environment. The learning procedure is as follows. The agent moves at *t_s_* to a new position in the input space, (*x_s_*, *y_s_*). After 50 ms at that position, the advocated action for that position, expressed as a 4-element column vector, is calculated as the product *a* (*x_s_*, *y_s_*) = *W_s_* (*t_s_* + 50)^T^ where *w* is the matrix of weights between striatal neurons and perceptrons, *s*(*t*) the row vector of low-pass filtered activity of striatal neurons at time *t* and T is the transpose operator. During the training phase, the trajectory functions are learned by adapting the weights between the striatal neurons and the perceptrons using a variant of the perceptron learning rule. This is calculated over the next 50 simulation steps (5 ms) according to the matrix equation:

$$\begin{array}{l} \Delta W=\sum_{k\;=\;1}^{50}\alpha (r\left(x_{\text{s}},y_{\text{s}}\right)/\left|r\left(x_{\text{s}},y_{\text{s}}\right)\right|\\ -a\left(x_{\text{s}},y_{\text{s}}\right)/\left|a\left(x_{\text{s}},y_{\text{s}}\right)\right|)s\left(t_{\text{s}}+50+0.1k\right) \end{array}$$

where α is the learning rate and *r* (*x*_s_, *y*_s_) the 4-element column vector indicating the correct movement according to the trajectory function. After a further 245 ms in that position (300 ms in total), the agent moves to a new position in space (*x*_s+1_, *y*_s+1_) according to the advocated action *a* (*x*_s_, *y*_s_). The procedure is repeated every 300 ms for a total of 3000 training steps.

### 4.4. Computational properties

#### 4.4.1. Performance

In order to assess the capability of the linear readouts to advocate the desired movements based only on the internal states of the striatal network (i.e., to assess the approximation property), we calculate the average difference over all testing steps, *t_i_*, between the normalized correct movement vector and the normalized movement vector advocated by the linear readouts (see Section 4.3):

(3) $$\text{avg}\left(r(t_{i})/\left|r(t_{i})\right|-a(t_{i})/\left|a(t_{i})\right|\right),\;t_{i}>t_{\text{training}}$$

The smaller the difference the higher the performance achieved by the linear readouts, which signifies a better piece-wise linear approximation and thus a better liquid state.

#### 4.4.2. Generalization

The ability to generalize a learned computational function to a new set of inputs, unseen throughout training is a very important feature of neural microcircuits.

In order to quantify the generalization capacity of our striatal microcircuit, we calculate the average difference over every testing position (*P* → (*x_s_*, *y_s_*)) between the normalized movement vector advocated by the linear readouts (see Section 4.3) and the normalized movement vector advocated from a slightly shifted position (P^*^ → (x^*^_s_, y^*^_s_)):

(4) $$\text{avg}\left(a(t_{i})/\left|a(t_{i})\right|-a{\left(t_{i}\right)}^{*}/\left|a{\left(t_{i}\right)}^{*}\right|\right),\;t_{i}>t_{\text{training}}$$

where the position P^*^ in the flat 2*D* surface, corresponding to *a*(*t_i_*)^*^, is chosen to lie at a randomly chosen angle (obtained from a uniform probability distribution in [0, 2π]), and at a randomly chosen distance (drawn from a Gaussian distribution centered 4 μm away, with a standard deviation of 4/3 μm) from the testing position.

The lower this difference, the higher the ability of the circuit to generalize beyond the training data, and thus use the same states to advocate distinct actions.

### Conflict of interest statement

The authors declare that the research was conducted in the absence of any commercial or financial relationships that could be construed as a potential conflict of interest.
