# Supplementary material for: Liquid computing on and off the edge of chaos with a striatal microcircuit
Source: Front Comput Neurosci. 2014 Nov 21;8:130. doi: 10.3389/fncom.2014.00130 (PMC4240071; doi:10.3389/fncom.2014.00130)
Supplement: Supplementary file 1 [file Table1.PDF]

Supplementary Material: Liquid computing on and off the edge of chaos with a striatal microcircuit

Carlos Toledo-Suárez [1,2,3], Renato Duarte [1,2,4,6] and Abigail Morrison [1,4,5*]

[1] *Bernstein Center Freiburg, Albert-Ludwig University of Freiburg, Freiburg im Breisgau, Germany*
[2] *Faculty of Biology, Albert-Ludwig University of Freiburg, Freiburg im Breisgau, Germany*
[3] *Department of Computational Biology, School of Computer Science and Communication, KTH Stockholm, Sweden*
[4] *Institute for Advanced Simulation (IAS-6) and Institute of Neuroscience and Medicine (INM-6), Jülich Research Centre and JARA Jülich, Germany*
[5] *Institute of Cognitive Neuroscience, Faculty of Psychology, Ruhr-University Bochum, Bochum, Germany*
[6] *Institute of Adaptive and Neural Computation, School of Informatics, University of Edinburgh, United Kingdom*

Correspondence*:
Abigail Morrison
Jülich Research Center and JARA,
Institute of Neuroscience and Medicine (INM-6) and Institute for Advanced Simulation (IAS-6),
Building 15.22, 52425 Jülich, Germany, a.morrison@fz-juelich.de

1 SUPPLEMENTARY TABLES

Table 1. Tabular description of network model.

**A: Model Summary**	
**Populations**	Input encoding cortical neurons (Cs), striatal medium spiny neurons (MSNs) and striatal fast spiking interneurons (FSIs)
**Connectivity**	Feed forward from Cs to MSNs and FSIs, feed forward from FSIs to MSNs, recurrent between MSNs
**Neuron model**	Leaky integrate-and-fire with exponential post-synaptic currents for Cs, leaky integrate-and-fire with exponential post-synaptic currents and multi-timescale adaptive threshold tuned for intrinsic bursting for MSNs and for fast spiking for FSIs
**Input**	Independent fixed-rate Poisson spike trains to Cs,<br>gaussian current profile to Cs
**Measurements**	Spike activity

**B: Populations**		
**Name**	**Elements**	**Size**
Cs	iaf psc exp neuron	50, 25 per axis
MSNs	mat2 iaf psc exp neuron	500
FSIs	mat2 iaf psc exp neuron	50

**C: Connectivity**			
**Name**	**Source**	**Target**	**Pattern**
$FF_{\mathrm{C-MSN}}$	Cs	MSNs	Random convergent, $C_{\mathrm{Ex}} \to 1$, weight $w_c J_{\mathrm{CMSN}}$, delay d_{C}
$FF_{\mathrm{C-FSI}}$	Cs	FSIs	Random convergent, $C_{\mathrm{Ex}} \to 1$, weight $w_c J_{\mathrm{CFSI}}$, delay d_{C}
INH_{FSI}	FSIs	MSNs	Random divergent, $1 \to C_{\mathrm{FSI}}$, weight $w_s J_{\mathrm{FSI}}$, delay d_{FSI}
INH_{MSN}	MSNs	MSNs	Random divergent, $1 \to C_{\mathrm{MSN}}$, weight $w_s J_{\mathrm{MSN}}$, delay d_{MSN}

**D: Neuron Models**	
**Name**	iaf_psc_exp neuron
**Type**	Leaky integrate-and fire with exponential post synaptic currents
**Subthreshold dynamics**	if $(t > t^* + \tau_{\mathrm{ref}})$ $\tau_{\mathrm{m}} \frac{dV}{dt} = -V + \frac{I_{\mathrm{syn}}(t)}{C_{\mathrm{m}}}$ else $V(t) = V_{\mathrm{reset}}$<br>$I_{\mathrm{syn}} = \sum_i \sum_{s \in S_i} w_i J_i I_{exp}(t-s-d)$<br>$I_{exp}(t) = e^{-t/\tau_{\mathrm{exp}}}$
**Spiking**	If $V(t-) < \Theta$ OR $V(t+) \geq \Theta$<br>1. set $t^* = t$<br>2. emit spike with time stamp t^*<br>Membrane potential reset:$V(t) = V_{\mathrm{reset}}$
**Threshold dynamics**	Fixed threshold
**Name**	mat2_psc_exp neuron (**Kobayashi et al.**, 2009)
**Type**	Leaky integrate-and-fire with exponential post-synaptic currents and multi-timescale adaptive threshold
**Subthreshold dynamics**	Same as iaf_psc_exp neuron
**Spiking**	Same as iaf_psc_exp neuron, without membrane potential reset
**Threshold dynamics**	$\Theta(t) = \sum_k H(t - t_k) + \omega, \qquad H(t) = \sum_{j=1}^{2} \alpha_j \exp(-t/\tau_j)$,

**E: Input**		
**Type**	**Target**	**Description**
**Poisson generator**	Cs	Independent for each neuron, rate ν_{back}, weight J_{back}
**Gaussian current profile**	Cs	$I_{\mathrm{Gauss}} \exp(-(i-x)^2/(2\sigma^2))$, per axis, where:<br>$i \in \{1, 2, \ldots, 25\}$ runs among Cs, $0 \leq x \leq 25$ tells the position on axis

**F: Measurements**
Low pass filtering of spikes from MSNs and FSIs

Table 2. Simulation parameters.

A: Connectivity		
**Name**	**Value**	**Description**
C_{Ex}	6	Number of outgoing feed-forward connections received by every striatal neuron from Cs
C_{FSI}	variable	Number of feed-forward connections from a FSI to MSNs found on a circular area centered on the FSI with radius R_{FSI}, chosen with uniform probability P_{FSI}
C_{MSN}	variable	Number of recurrent connections from a MSN to other MSNs found on a circular area centered on it with radius R_{MSN}, chosen with Gaussian probability, such that the total probability a standard deviation away is fixed to P_{MSN}
J_{CMSN}	100 pA	Amplitude of excitatory connection from a cortical neuron to a MSN
J_{CFSI}	$1.7\times$ pA	Amplitude of excitatory connection from a cortical neuron to a FSI
J_{FSI}	variable	Amplitude of inhibitory connection from a FSI to a MSN, taken from a random uniform distribution on $[-480,-50]$ pA (**Koos et al.**, 2004)
J_{MSN}	variable	Amplitude of inhibitory connection from a MSN to a MSN, taken from a random uniform distribution on $[-90,-10]$ pA (**Koos et al.**, 2004)
d_C	1 ms	Synaptic transmission delay from Cs to MSNs and FSIs
d_{FSI}	1 ms	Synaptic transmission delay from FSIs to MSNs
d_{MSN}	2 ms	Synaptic transmission delay from MSNs to MSNs
R_{FSI}	0.1 mm	Radius for circular area around FSI to make a connection with MSN (**Planert et al.**, 2010)
R_{MSN}	1 mm	Radius for circular area around MSN to make a connection with another MSN
P_{FSI}	0.74	Uniform probability to make a connection between FSI and MSN (**Planert et al.**, 2010)
P_{MSN}	0.2	Probability within a Gaussian standard deviation away from MSN to make a connection with another MSN (**Planert et al.**, 2010)

B: Neuron Model		
**Name**	**Value**	**Description**
τ_{mC}	10 ms	C membrane time constant
C_{mC}	250 pF	C membrane capacitance
Θ_C	-55 mV	C Fixed firing threshold
V_{0C}	-70 mV	C resting potential
$V_{\mathrm{reset}C}$	V_{0C}	C reset potential
τ_{refC}	2 ms	C absolute refractory period
τ_{mM}	5 ms	MSN membrane time constant
C_{mM}	200 pF	MSN membrane capacitance (**Gertler et al.**, 2008)
V_{0M}	-58 mV	MSN resting potential
τ_{refM}	1 ms	MSN absolute refractory period
τ^{+}_{sM}	0.2 ms	MSN time constant of post-synaptic excitatory currents
τ^{-}_{sM}	2 ms	MSN time constant of post-synaptic inhibitory currents
α_{1M}	7.5 mV	Weight of MSN multi-timescale adaptive threshold first time constant
α_{2M}	1.5 mV	Weight of MSN multi-timescale adaptive threshold second time constant
ω_M	19 mV	MSN multi-timescale adaptive threshold resting value

B: Neuron Model		
**Name**	**Value**	**Description**
τ_{mF}	$5\,\mathrm{ms}$	FSI membrane time constant
C_{mF}	$500\,\mathrm{pF}$	FSI membrane capacitance
V_{0F}	$-68\,\mathrm{mV}$	FSI resting potential
τ_{refF}	$2\,\mathrm{ms}$	FSI absolute refractory period
τ^{+}_{sF}	$0.3\,\mathrm{ms}$	FSI time constant of post-synaptic excitatory currents
τ^{-}_{sF}	$2\,\mathrm{ms}$	FSI time constant of post-synaptic inhibitory currents
α_{1F}	$10\,\mathrm{mV}$	Weight of FSI multi-timescale adaptive threshold first time constant
α_{2F}	$0.2\,\mathrm{mV}$	Weight of FSI multi-timescale adaptive threshold second time constant
ω_F	$10\,\mathrm{mV}$	FSI multi-timescale adaptive threshold resting value

C: Input		
**Name**	**Value**	**Description**
ν_{back}	$10^4\,\mathrm{Hz}$	Background independent Poisson rate to Cs
J_{back}	$20\,\mathrm{pA}$	Amplitude of background independent Poisson process to Cs
I_{Gauss}	$1\,\mathrm{nA}$	Maximum amplitude for Gaussian current profile
σ	2	Standard deviation of Gaussian current profile

D: Supervised learning	
**Parameter**	**Value**
**Time step**	$0.1\,\mathrm{ms}$
**Learning rate**	0.1
**Runtime per step**	$300\,\mathrm{ms}$
**Training samples per step**	50 after $50\,\mathrm{ms}$
**Time in current position to take sample for advocated action**	$50\,\mathrm{ms}$
**Low pass filter decay**	$30\,\mathrm{ms}$

REFERENCES

Gertler, T. S., Chan, C. S., and Surmeier, D. J. (2008), Dichotomous anatomical properties of adult striatal medium spiny neurons, *The Journal of Neuroscience*, 28, 43, 10814–10824, doi:10.1523/JNEUROSCI.2660-08.2008

Kobayashi, R., Tsubo, Y., and Shinomoto, S. (2009), Made-to-order spiking neuron model equipped with a multi-timescale adaptive threshold., *Frontiers in computational neuroscience*, 3, July, 9, doi:10.3389/neuro.10.009.2009

Koos, T., Tepper, J. M., and Wilson, C. J. (2004), Comparison of ipscs evoked by spiny and fast-spiking neurons in the neostriatum, *The Journal of Neuroscience*, 24, 36, 7916–7922, doi:10.1523/JNEUROSCI.2163-04.2004

Planert, H., Szydlowski, S. N., Hjorth, J. J. J., Grillner, S., and Silberberg, G. (2010), Dynamics of synaptic transmission between fast-spiking interneurons and striatal projection neurons of the direct and indirect pathways, *The Journal of Neuroscience*, 30, 9, 3499–3507, doi:10.1523/JNEUROSCI.5139-09.2010
